# Enhancing statistical power in temporal biomarker discovery through representative shapelet mining

**DOI:** 10.1093/bioinformatics/btaa815

**Published:** 2020-12-29

**Authors:** Thomas Gumbsch, Christian Bock, Michael Moor, Bastian Rieck, Karsten Borgwardt

**Affiliations:** Department of Biosystems Science and Engineering, ETH Zurich, Basel 4058, Switzerland; SIB Swiss Institute of Bioinformatics, Lausanne 1015, Switzerland; Department of Biosystems Science and Engineering, ETH Zurich, Basel 4058, Switzerland; SIB Swiss Institute of Bioinformatics, Lausanne 1015, Switzerland; Department of Biosystems Science and Engineering, ETH Zurich, Basel 4058, Switzerland; SIB Swiss Institute of Bioinformatics, Lausanne 1015, Switzerland; Department of Biosystems Science and Engineering, ETH Zurich, Basel 4058, Switzerland; SIB Swiss Institute of Bioinformatics, Lausanne 1015, Switzerland; Department of Biosystems Science and Engineering, ETH Zurich, Basel 4058, Switzerland; SIB Swiss Institute of Bioinformatics, Lausanne 1015, Switzerland

## Abstract

**Motivation:**

Temporal biomarker discovery in longitudinal data is based on detecting reoccurring trajectories, the so-called *shapelets*. The search for shapelets requires considering all subsequences in the data. While the accompanying issue of multiple testing has been mitigated in previous work, the redundancy and overlap of the detected shapelets results in an *a priori* unbounded number of highly similar and structurally meaningless shapelets. As a consequence, current temporal biomarker discovery methods are impractical and underpowered.

**Results:**

We find that the pre- or post-processing of shapelets does not sufficiently increase the power and practical utility. Consequently, we present a novel method for temporal biomarker discovery: Statistically Significant Submodular Subset Shapelet Mining (S5M) that retrieves short subsequences that are (i) occurring in the data, (ii) are statistically significantly associated with the phenotype and (iii) are of manageable quantity while maximizing structural diversity. Structural diversity is achieved by pruning non-representative shapelets via submodular optimization. This increases the statistical power and utility of S5M compared to state-of-the-art approaches on simulated and real-world datasets. For patients admitted to the intensive care unit (ICU) showing signs of severe organ failure, we find temporal patterns in the sequential organ failure assessment score that are associated with in-ICU mortality.

**Availability and implementation:**

S5M is an option in the python package of S3M: github.com/BorgwardtLab/S3M.

## 1 Introduction

In hospitals, critically ill patients are transferred to intensive care units (ICUs) and are subjected to increased intensity of monitoring and care. The sequential organ failure assessment score (SOFA) describes the severity of a patient’s organ dysfunction where a high score is associated with high in-ICU mortality (Singer et al., 2016; Vincent et al., 1996). Recently, there have been a growing number of publicly available critical care databases (Hyland *et al.*, 2020; Johnson *et al.*, 2016; Pollard *et al.*, 2018) recording patient variables, clinical actions and patient outcomes. In clinical practice, the SOFA score is assessed in 24 h intervals or more (Ferreira *et al.*, 2001; Tee *et al.*, 2018). The high resolution of critical care databases, however, makes more frequent evaluations of the SOFA scores possible. In this work, we mine critical care databases for statistically significant temporal patterns to provide additional information and assistance to clinicians in recognizing and interpreting clinical data.

The natural data mining choice for this task are *shapelets* (Ye and Keogh, 2009)—typically short subsequences that yield competitive classification accuracy given the class label (i.e. the phenotype) of the time series. For the biomedical domain, however, it is important to ensure the interpretability of shapelets in terms of a *P*-value, as they might be novel biomarkers. Therefore, Bock *et al.* (2018) employ association mapping, which is fundamentally different to classification in that statistical significance is ensured. Nevertheless, statistically significant shapelet mining is fundamentally underpowered because it does not account for redundant or structurally meaningless (e.g. flat) shapelets. This may become a problem for downstream applications, for example when implementing a medical assistance system based on thousands of highly similar *significant* shapelets: monitoring many features increases the risk of a clinician being overwhelmed by alarms, alerts and notifications—which is among the top 10 health technology hazards in 2020 (ECRI, 2019). Therefore, we build a method maximizing statistical power and representativeness of the shapelets by searching for a set of statistically significant shapelets with maximal structural diversity of manageable size: Statistically Significant Submodular Subset Shapelet Mining (S5M).

S5M selects shapelets using Tarone’s multiple test procedure (Tarone, 1990) and classifies them as (non-)representative by optimizing a submodular mixture objective function (Libbrecht *et al.*, 2018). Instead of pre- or post-processing shapelets to select a representative subset, S5M declares non-representative shapelets as non-testable which leads to an increase in statistical power and runtime compared to any two-step alternative. To declare statistical significance and representativeness simultaneously, we introduce a new iterative solver for submodular optimization problems. Therefore, S5M returns a structurally more diverse and useful shapelet set than the state-of-the-art, which is confirmed on simulation data and on patient data from the MIMIC-III database (Johnson *et al.*, 2016). We also discover novel interpretable biomarkers in the sequential organ failure assessment (SOFA) score of patients associated with in-ICU mortality on the eICU database (Pollard *et al.*, 2018).

## 2 Related work

The search for a structurally diverse subset of manageable size can be summarized as finding a set of sequences that is *minimally redundant* and whose elements exhibit *maximum coverage*. The traditional approach to extract a *representative subset of an itemset* is described by the threshold algorithm of Hobohm *et al.* (1992): (i) initialize an empty set of representatives. (ii) Iteratively fill this set with sequences whose similarity to all currently representative sequences is less than a given threshold. The drawbacks of not being able to trade off redundancy with coverage and having no approximation guarantees of the threshold algorithm are mitigated by solving a submodular mixture objective function, which leads to the successful selection of a representative training set in machine learning (Lin and Bilmes, 2009; Wei *et al.*, 2015) and prioritization of sequence data experiments (Libbrecht *et al.*, 2018; Yilmaz *et al.*, 2019).

For discovering *representative subsequences in time series*, Imani *et al.* (2018) introduce the concept of ‘snippets’, a greedy search over all non-overlapping time series subsequences that rewards coverage by considering the MPdist (Gharghabi *et al.*, 2018) between a representative candidate and all-time series. Similarly, in Mueen *et al.* (2009), the authors extract representative subsequences, or ‘motifs’, by rewarding fidelity of coverage. The caveat of these approaches is that (i) they are not exact, in the sense that only non-overlapping subsequences are considered, and (ii) while ‘snippets’ considers a mixture objective function, it is not possible to *weight* one objective more important than the other.

Traditionally, shapelets serve as a frequency-based feature extraction approach for time series subsequences enabling competitive classification accuracy (Karlsson *et al.*, 2016). Due to the enumeration problem of time series subsequences of all lengths (Rakthanmanon *et al.*, 2012), there have been efforts in constraining the shapelet search, e.g. by requiring structural diversity of the retrieved shapelets. In Fang *et al.* (2018) and in Ghalwash *et al.* (2013), easy to interpret subsequences are extracted by optimizing for classification accuracy. However, statistical validation is not inherent to the shapelets retrieved in the classification setting and has to be done a posteriori via a Bonferroni correction, which is too conservative and results in no significant associations (Pržulj, 2019, Chapter 8).

Statistical validation of shapelets is tackled in Bock *et al.* (2018), which allows for meaningful interpretation of the features in terms of *P*-values. Compared to the approaches for discovering interpretable shapelets, however, there is no way of controlling the cardinality of the set of shapelets returned and thus many redundant or structurally meaningless shapelets may appear. Therefore, applications of statistically significant shapelet mining will require pre- or post-processing of shapelets and thus suffer from low statistical power (Hyland *et al.*, 2020).

**Figure:**
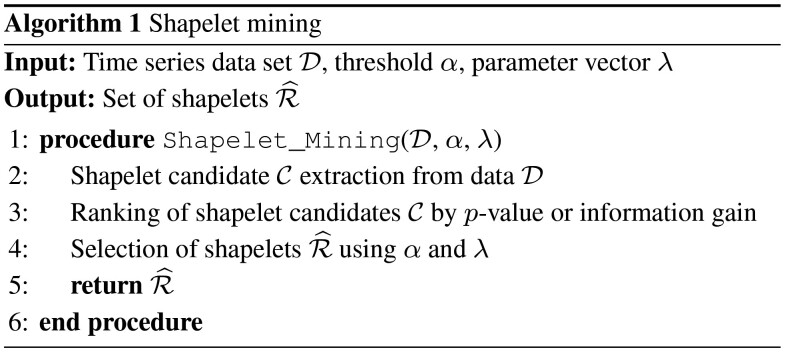


## 3 Materials and methods

### 3.1 Shapelet mining

We assume a dataset $D={\left\{(t^{i},y^{i})\right\}}_{0<i\leq n}$ of *n* time series and class labels for each time series, where *m_i_* is the length of time series *i*, i.e. $t^{i}=(t_{1}^{i},\ldots ,t_{m_{i}}^{i})$, and $y^{i}\in \{0,1\}$ is the binary class label of *t^i^*. Since shapelets are time series subsequences that can be different lengths, the comparison of shapelets requires a distance measure for sequences of unequal length. The traditional distance measure for shapelets is the minimum Euclidean distance between the shorter sequence $r=(r_{0},\ldots ,r_{m_{r}})$ and the longer sequence $s=(s_{0},\ldots ,s_{m_{s}})$ (i.e. $m_{s}\geq m_{r}$) over all possible alignments of *r* on *s* (Ye and Keogh, 2009):

(1) $$\mathrm{dist}(r,s)=\underset{0\leq j<|s|-|r|}{\min}{\left(\sum_{k=0}^{k<|r|}{\left(s_{j+i}-r_{k}\right)}^{2}\right)}^{1/2}.$$

A shapelet is a tuple of two items: a time series subsequence and a distance threshold (s,θ). A shapelet predicts a class label ${\hat{y}}^{i}$ for a time series *t^i^* if

(2) $${\hat{y}}_{\left(s,\theta \right)}^{i}=\mathrm{dist}(s,t^{i})\leq \theta .$$

In general, shapelet mining approaches consist of the following setup (see Algorithm 1): the inputs to the method are D, a dataset of time series *t^i^* with a class label *y^i^* for each time series, *α*, a threshold on the shapelet selection, and λ, a vector of parameters to the method. First, in Line 2, shapelet candidates C are extracted from the time series. Second, in Line 3, a quality measure gives a ranking P of the shapelet candidates. The quality of a shapelet is usually determined by a measure of statistical dependence between the shapelet occurrence and the class label, i.e. by information gain (Ye and Keogh, 2009). Finally, in Lines 5 and 6, a selection of the shapelets is returned by applying the threshold *α*.

### 3.2 Statistically significant shapelet mining

In the following, we briefly describe the association mapping approach of Bock *et al.* (2018). This means Algorithm 1 is concretized in the following way: First, as input, we specify the family-wise error rate (FWER), which is the probability of generating one or more false positives that we set to α=0.05 for all experiments. Next, in Line 2, shapelet candidates are extracted with a sliding window method of variable width *w*. In Line 3, Bock *et al.* (2018) use the minimum *P*-value over all thresholds based on a $\chi^{2}$-test (Pearson, 1900) as a measure to rank shapelets, i.e.

(3) $$p_{\text{min}}(s)=\underset{\theta \in \{\mathrm{dist}(s,t)|t\in D\}}{\text{min}}p_{\chi^{2}}\{({\hat{y}}_{\left(s,\theta \right)}^{i},y^{i})|0<i\leq n\}.$$

In Line 4, statistically significant shapelet mining selects significant shapelets with minimum *P*-value smaller than the multiple-testing corrected FWER, i.e. $\hat{R}=\{s|p_{\text{min}}(s)<\hat{\delta },s\in C\}$. The set of statistically significant shapelets therefore depends on $\hat{\delta }$, the multiple-testing corrected significance threshold that is computed using the distribution of the minimum *P*-values of all shapelets, P, and the target FWER *α*.

#### 3.2.1 Correcting for multiple testing

Since the search for shapelets requires testing all subsequences of the dataset D for all possible thresholds, it results in an enormous multiple testing problem (Shaffer, 1995) that requires controlling for the FWER *α*. Correcting *α* without any further assumptions on the test statistic or the distribution of the null hypotheses is traditionally accomplished with a Bonferroni correction (Bonferroni, 1936), which divides *α* by the number of statistical tests conducted to assess the *P*-value of each shapelet (there are *n* thresholds per shapelet in the set on candidate subsequences C), i.e.

(4) $$\hat{\delta_{\text{BF}}}=\frac{\alpha }{|C|n}$$

Bock *et al.* (2018) observe the Bonferroni correction can be too conservative to detect statistically significant shapelets. Instead, they propose to leverage Tarone’s method (Tarone, 1990) for assessing the statistical significance of a shapelet. The insight of Tarone is that for discrete test statistics (e.g. Fisher’s exact test or a $\chi^{2}$ test), there is a minimum attainable *P*-value that a pattern can attain based on its frequency. All patterns *s* whose minimum attainable *P*-value $p_{\text{min}}(s)$ is larger than a continuously decreasing significance threshold are deemed *untestable* as they can never contribute to the FWER. Hence, the significance threshold is obtained as

(5) $${\hat{\delta }}_{\text{tar}}=\frac{\alpha }{\left|\{s|p_{\text{min}}(s)<{\hat{\delta }}_{\text{tar}},s\in C\}\right|}.$$

**Figure:**
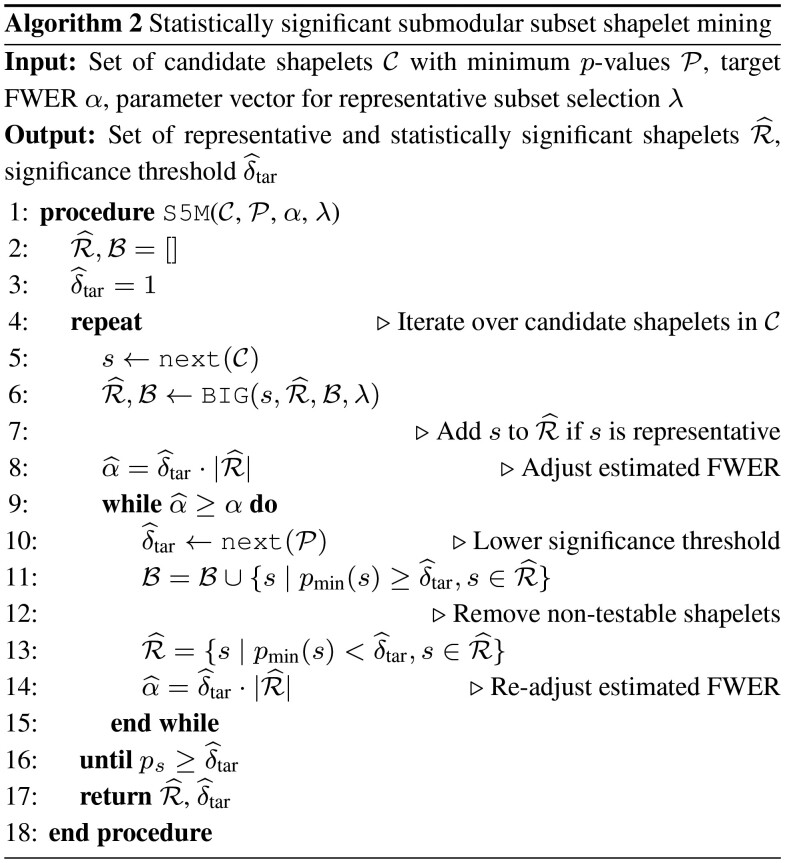


### 3.3 Statistically significant submodular subset shapelet mining (S5M)

This section describes our proposed approach, S5M, where an iterative selection of representative shapelet candidates prunes non-representative shapelets before adjusting for multiple testing via Tarone’s method. For now, we assume the classification of shapelets into representative and non-representative to be known; the full description of the *bidirectional iterative greedy* algorithm (BIG) to optimize the submodular mixture objective function can be found in Section 3.5.1 and Algorithm 3.

In S5M (Algorithm 2), the set of statistically significant and representative shapelets $\hat{R}$ and the set of non-representative and non-testable shapelet candidates B are initialized to the empty set in Line 2. In Line 3, the Tarone significance threshold is initialized to one, because no shapelets have been selected yet. Next, we iterate over all shapelet candidates and their minimum *P*-values. In Line 5, the next shapelet candidate *s* will always appear in the order of their minimum *P*-value (we process candidates from lower minimum *P*-values to higher minimum *P*-values). In Line 6, we apply the representative subset search with BIG (Algorithm 3) to check whether *s* is a representative shapelet, given the previously processed candidates. If *s* is added to the set of representatives $\hat{R}$, the estimated FWER $\hat{\alpha }$ will increase in Line 8. If the estimated FWER is higher than the desired error rate, the Tarone significance threshold will be decreases (Line 9) and the non-testable shapelets from $\hat{R}$ will be removed (Line 11) until the estimated FWER is smaller or equal to the desired error rate (Lines 13 and 14). The shapelets removed from $\hat{R}$ are assigned to the set of non-representative and non-testable shapelets B in Line 10. Note that we check whether the minimum *P*-value of the candidate *s* is above the current significance threshold ${\hat{\delta }}_{\text{tar}}$ in Line 13 and could prune many of the candidate shapelets by exiting the outer loop of Line 4 before C is empty. Moreover, since we process the candidates from the smallest to largest minimum *P*-values and ${\hat{\delta }}_{\text{tar}}$ is monotonically decreasing, the number of statistically significant shapelets does not increase when lowering the threshold. Finally, in Line 17, the set of representative and statistically significant shapelets are returned.

By pruning both non-representative and non-testable shapelets simultaneously, S5M is expected to have more power compared to a two-step approach. A feature selection approach, which extracts representatives in Line 2 of Algorithm 1, will be less sensitive, because the representatives capture the full dataset and not the diversity that is due to the class label. In a post-processing approach, the Tarone significance threshold is too conservative, because representatives are selected *after* declaring significance. The large number of non-representative, statistically significant shapelets will lower the power of this approach. Both two-step comparison partners are also contrasted to S5M experimentally in Sections 4.2 and 4.3.

### 3.4 Clustering of shapelets

For extracting representative shapelets, a similarity measure between unequal length subsequences is required. Since shapelets come with a distance measure, we compute the similarity between shapelet candidates *r* and *s* with the transformation

(6) $$\mathrm{sim}(r,s)={\left(1+\mathrm{dist}(r,s)\right)}^{-1}.$$

Note that the traditional shapelet distance measure (as defined in Equation 1) is not a metric because the triangle inequality does not hold. This poses a problem for clustering shapelets, because clustering groups similar items: When clustering with a similarity measure constructed from the minimum distance, two highly dissimilar items might falsely end up in the same cluster because they can share close similarity to a third item. Another reason against clustering shapelets is the additional challenge of specifying a procedure for selecting a representative from each cluster. A third reason against clustering shapelets is that shapelets are overlapping time series subsequences where naive application of *k*-means clustering results in meaningless clusters (Keogh and Lin, 2005).

On a larger scale, significant shapelet mining can be viewed as an instance of significant pattern mining (Terada *et al.*, 2013), which also faces the challenge of an uncontrollably large number of statistically significant patterns, e.g. for testing single nucleotide polymorphism (SNP) interaction in genome wide association studies (GWAS) (Llinares-López *et al.*, 2019). The problem is mitigated by clustering items by their overlap and only returning the item with the lowest *P*-value (Papaxanthos *et al.*, 2016). For GWAS, the SNP location (e.g. the gene in which the SNP lies) has an interpretation. For shapelets, however, the point in time of the originating time series has no associated biological interpretation.

In contrast to clustering, representative subset selection extracts individual items chosen to represent a larger set which has fewer requirements, e.g. even symmetry is not needed for the underlying distance measure.

**Figure:**
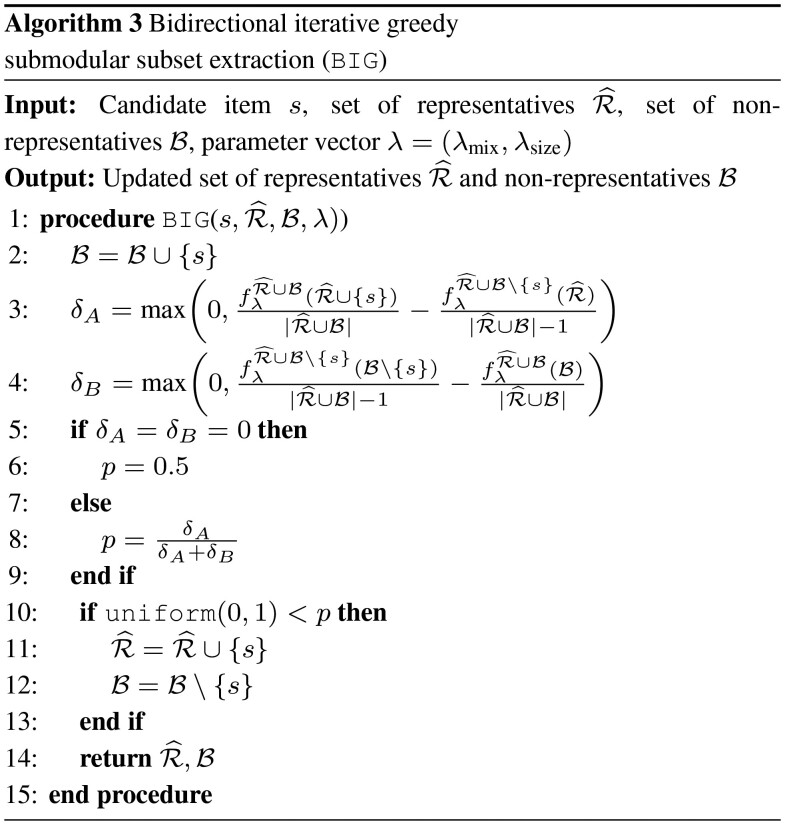


### 3.5 Submodular subset selection

Submodularity is a property of a function $f^{S}:2^{S}\to \mathbb{R}$ acting on a finite set S. Following Fujishige (2005), *f* is submodular if and only if, for a subset R and B where R⊆B⊂S and an element s∈S with s∉B
 

(7) $$f^{S}(R\cup \{s\})-f^{S}(R)\geq f^{S}(B\cup \{s\})-f^{S}(B).$$

Intuitively, submodularity of functions on sets is the analogue of convexity of continuous functions. If a function is submodular, it can be optimized with standard approximation algorithms that find a solution that is guaranteed to be within a constant factor of the optimal solution (Nemhauser *et al.*, 1978).

Libbrecht *et al.* (2018) introduce a submodular subset quality measure for choosing a representative subset of protein sequence data. We chose to apply their approach due to the mathematical similarities between sequence and time series data, in contrast to other cut-based approaches where we would need to first construct a graph, as in Yilmaz *et al.* (2019). The mixture objective by Libbrecht *et al.* (2018) is weighted by a parameter $\lambda_{\text{mix}}$. A representative subset R of a set S is defined as

1. maximizing the similarity between every item in full set to the most similar item in the representative set, called the *facility-location*
 
  (8) $$f_{fl}^{S}(R)=\frac{1}{\left|S\right|}\sum_{s\in S}\underset{r\in R}{\max}\{\mathrm{sim}(s,r)\}$$
2. and minimizing the pairwise similarity between items of the representative set, called the *sum-redundancy*
 
  (9) $$f_{sr}^{S}(R)=\sum_{a,b\in S}\mathrm{sim}(a,b)-\sum_{r_{1},r_{2}\in R}\mathrm{sim}(r_{1},r_{2}).$$

Both functions are submodular and thus the mixture objective is also submodular. If the target number of items in the representative subset is unknown, the objective is extended by a regularization term penalizing larger subsets with a parameter $\lambda_{\text{size}}$. In our notation, we subsume both parameters by denoting $\lambda =(\lambda_{\text{mix}},\lambda_{\text{size}})$.

To summarize, the quality of a representative subset R of a larger set S is given as

(10) $$f_{\lambda }^{S}(R)=\lambda_{\text{mix}}f_{fl}^{S}(R)+(1-\lambda_{\text{mix}})f_{sr}^{S}(R)+\lambda_{\text{size}}|R|.$$

It has been shown by Libbrecht *et al.* (2018) that the setting of $\lambda_{\text{mix}}\in (0.25,0.75)$ does not significantly change the accuracy of the algorithm and therefore $\lambda_{\text{mix}}=0.5$ is set in all cases. $\lambda_{\text{size}}=1$ is chosen by us because it works well in a diverse range of datasets.

#### 3.5.1 Submodular optimization with BIG

Discovering the optimal representative subset requires submodular optimization of the mixture objective in Equation 3.5. Optimizing via the greedy algorithm means sequentially adding the item that decreases the objective function as much as possible, requiring a search over the full dataset for each element, i.e. *n*^2^ computations of the objective function. Computing the objective once requires computational complexity of $O(n^{2})$ for *n* items because of the double sum in the sum-redundancy objective. That means extracting representatives via the greedy algorithm has a worst-case complexity of $O(n^{4})$. The bidirectional greedy algorithm of Libbrecht *et al.* (2018) improves upon the computational complexity by iterating once over the full dataset while maintaining a *growing* set $\hat{R}$ and a *shrinking* set B. Items are assigned stochastically to the sets with probabilities proportional to the marginal gains in objective functions. The *bidirectional greedy* algorithm therefore requires a worst-case computational complexity of $O(n^{3})$ for *n* items.

However, the *bidirectional greedy* algorithm cannot be used to select shapelets within Tarone, because the size and the elements of the *shrinking* set B are not known in advance due to ${\hat{\delta }}_{\text{tar}}$ changing after each iteration. The key difference to the proposed bidirectional iterative greedy (BIG) algorithm is that B has to be initialized to the empty set and contains the items that are declared *non-testable* because they are already represented by another item. Consequently, for every shapelet candidate in S5M, BIG (Algorithm 3) can be called in Line 6 of Algorithm 2. BIG declares this item *s* as either representative (and *s* is added to $\hat{R}$) or as non-representative (and *s* is not removed from B).

Specifically, Algorithm 3 takes the previously returned sets $\hat{R}$ and B, the parameters λ, and the new item to be considered *s* as input. In Line 2, *s* is added to B. Then, the relative increase in the objective *f* of either adding *s* to $\hat{R}$ or removing *s* from B is computed in Lines 3 and 4. Because the size of the full set changes, computing the objectives requires normalization to the current set size, i.e. $\left|\hat{R}\cup B\right|$ or $|\hat{R}\cup B|-1$. We then compute a value *P* that is proportional to the respective change in the objective function in Line 8. If *P* would be invalid, it is set to 0.5 (Lines 5 to 7). In Lines 10 to 14, we use *P* as the probability for adding *s* to $\hat{R}$ and removing *s* from B. Both sets are returned in Line 14.

BIG requires the computation of the objective four times at each iteration, which has a computational complexity of $O(n^{2})$ per iteration. However, in contrast to the greedy or the bidirectional greedy algorithm, we note that the facility-location and the sum-redundancy can be updated from the value of the previous iteration. At iteration *i *+* *1 with ${\hat{R}}^{i+1}$ in the role of R in Equation 3.5, assume ${\hat{R}}^{i+1}={\hat{R}}^{i}\cup \{s^{i}\}$ and $B^{i+1}=B^{i}\cup \{s^{i+1}\}\setminus \{s^{i}\}$; in other words, at iteration *i*, *s^i^* was added to ${\hat{R}}^{i}$. Then,

(11) $$f_{\text{fl}}^{i+1}=f_{\text{fl}}^{i}+\underset{r\in {\hat{R}}^{i}\cup \{s^{i}\}}{\max}\mathrm{sim}(s^{i+1},r)$$

and

(12) fsri+1=fsri+∑a∈R^i∪Bi∪{si+1}sim(a,si+1)−∑r∈R^i∪{si}sim(r1,si).

Similar equations can be constructed for *s^i^* is remaining in $B^{i}$ and $B^{i+1}$ in the role of R. This eliminates the double sums in the execution of BIG and reduces the computational complexity of Algorithm 3 to *O*(*n*) per iteration, i.e. $O(n^{2})$ for S5M. The quantitative difference between the iterative version of BIG and the two algorithms proposed by Libbrecht *et al.* (2018) is investigated in Section 4.1.

## 4 Experiments

### 4.1 Simulation results for BIG

**Fig. 1.**
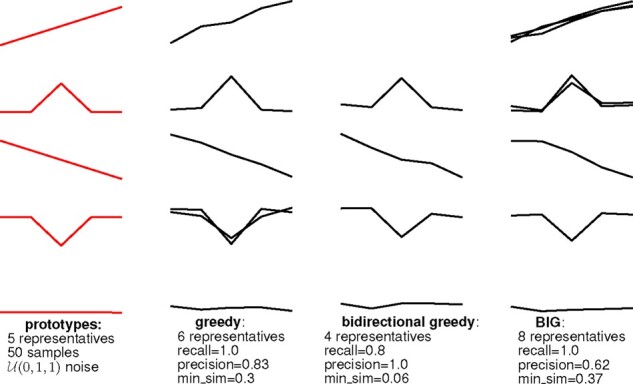
An illustrative example of how the three submodular optimization algorithms extract time series representatives (noise and sample size fixed). Precision and recall are computed using the ground truth representatives shown in red. Also shown is min_sim, the minimum similarity to the closest ground truth shapelet—the lower the min_sim, the higher the greatest error of the method

Section 3.5.1 proposes a novel submodular subset optimization algorithm—the *bidirectional iterative greedy* (BIG) algorithm. Here, we compare BIG to its state-of-the-art comparison partners, the greedy and the bidirectional greedy algorithm on simulation datasets. The datasets consist of a varying number of sequences that are built from five ground truth prototype sequences that are overlaid with uniform noise. Figure 1 shows the prototypes and the retrieved submodular subset. Here, the greedy algorithm (left) finds all ground truth representatives but has a high maximum error by achieving a low minimum similarity to the closest ground truth shapelet. The bidirectional greedy algorithm (middle) does not find all ground truth representative sequences whereas the bidirectional iterative greedy algorithm (right) finds too many sequences.

**Fig. 2.**
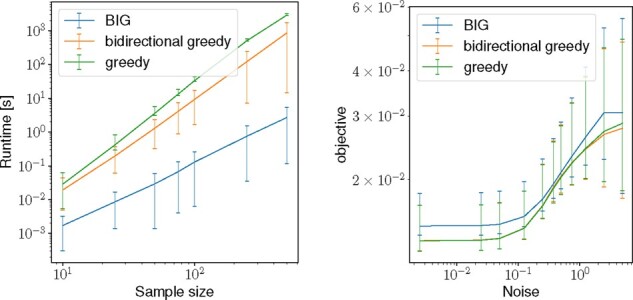
Comparison of three submodular optimization algorithms on a simulation dataset consisting of five ground truth prototype representatives (shown in red in Fig. 1). Left: the runtime in seconds (*y*-axis) when varying the number of replicates per prototype (*x*-axis). Right: the value of the objective function (*y*-axis) varying the uniform noise added to the prototype (*x*-axis)

To quantitatively compare the three algorithms, we vary logarithmically *n*, the number of sequences generated from the same representative prototype n∈{2,…,100} and *σ*, the uniform noise added to the sequence σ∈{0.01,…,20}. For each simulation, we create five repetitions each with a different randomization. From the right-hand size of Figure 2, we can observe a marginal difference in the objective value between the iterative and the other two algorithms for high levels of noise. At the same time, the left-hand side of Figure 2 shows the runtime comparison varying the sample size, where we can validate that the *bidirectional iterative greedy* algorithm (Algorithm 3) is multiple orders of magnitude faster compared to the non-iterative comparison partners. Note that all three algorithms scale in practice much better than their worst-case computational complexity may suggest – this is due to the very conservative bound on the complexity for computing the objective function, where we assumed the worst case of $|\hat{R}|=n$. We conclude that the proposed *bidirectional iterative greedy* algorithm BIG is a valid and faster alternative to both greedy algorithms. Unless stated otherwise, we use BIG for representative subset selection in all experiments.

### 4.2 Simulation results for S5M

This work proposes S5M for the extraction of representative and statistically significant shapelets. In the following, we validate that the proposed S5M method has higher statistical power compared to its alternative flavors discussed in the method section on datasets where the ground truth representative shapelets are known. Each simulation consists of five known ground truth shapelets R that are injected into the sequences of length *m *=* *20. The five prototype shapelets in R are the same as in Section 4.1 (Fig. 1 left), the control sequences consist of uniform noise. We create several instances of the dataset by varying the sample size n∈{100,200,500} and the variance of uniform noise σ∈{1,2,5}. For each simulation, we create 5 repetitions with a different randomization. We compare the following five algorithms for extracting representative statistically significant shapelets:

1. Feature Selection Tarone: After candidate extraction, representatives are selected from the candidates, irrespective of the class label. As a second step, statistically significant shapelet mining via Tarone’s multiple test procedure is performed.
2. Bonferroni submodular: As a first step, statistically significant shapelets are extracted using a Bonferroni significance correction (Equation 4). As a second step, representatives are extracted from the set of statistically significant shapelets.
3. Tarone submodular: As a first step, statistically significant shapelets are extracted using Tarone’s multiple test procedure. Then, representatives are selected via BIG.
4. S5M threshold: Representative and statistically significant shapelets are extracted simultaneously as in S5M where ‘representativeness’ is determined with the threshold method. The threshold is set to a uniform random value between 0 and 1.
5. S5M: Representative and statistically significant shapelets are extracted simultaneously as described in Section 3.3.

Note that in this experiment we follow the standard time series subsequences mining approach of providing the ground truth subsequence length to all algorithms (Rakthanmanon *et al.*, 2012); for all real-world data experiments in the subsequent sections, we allow a range of possible shapelet lengths.

Each method returns a number of shapelets $\hat{R}$ which we call *retrieved shapelets*. $\hat{R}$ can be evaluated in terms of precision and recall, by mapping each retrieved shapelet in $\hat{R}$ to its most similar ground truth shapelet in R. The number of correctly detected shapelets *k_d_* is the size of the image of that map. Shapelet recall is the number of correctly detected shapelets over the number of ground truth shapelets. Shapelet precision is the number of correctly detected shapelets over the number of retrieved shapelets. The F1 score is the harmonic mean of precision and recall. To be precise,

(13) $$\text{recall}=\frac{k_{d}}{\left|R\right|}\;,\;\text{precision}=\frac{k_{d}}{\left|\hat{R}\right|}$$

 

(14) $$F1\;\text{score}=\frac{2\cdot \text{precision}\cdot \text{recall}}{\text{precision}+\text{recall}}$$

**Fig. 3.**
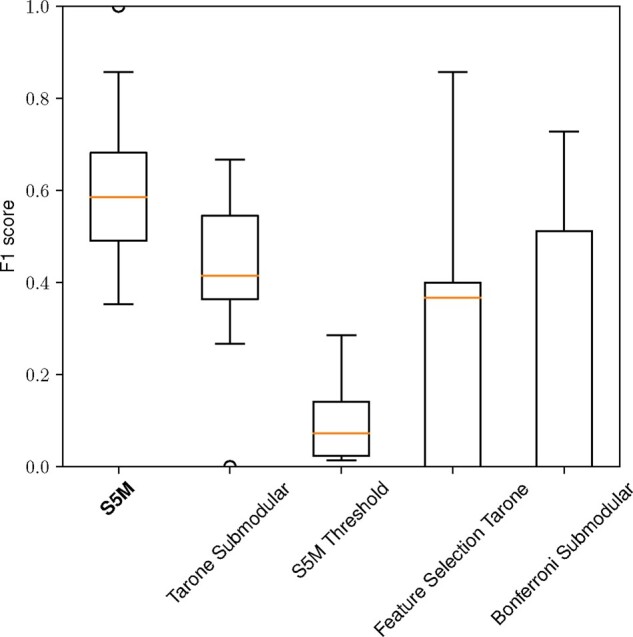
Comparing the F1 score of alternative strategies for extracting representative statistically significant shapelets on a simulation dataset varying the noise added to the prototype sequence and number of time series. S5M significantly outperforms its competitors (*P *<* *0.005 with Welch’s *t*-test)

Figure 3 shows the results of this experiment. First, we observe the S5M threshold approach having a very low F1 score. Investigating precision and recall showed this is due to a very high number of shapelets and thus very low precision. The variance being small, we hypothesize the large error is not due to an incorrect choice of the threshold, but due to the underlying method being based on clustering and not on representative subset selection. Second, the error of the Bonferroni submodular approach and the Feature selection Tarone approach vary more than for the other two methods. Investigating the components of the F1 score, we find the Feature selection Tarone approach has low recall in most cases, because it suffers from the representative selection being the first step. This means the representatives capture the full dataset and not the diversity that is due to the class label. The Bonferroni submodular approach has a low recall because few shapelets are returned due to the Bonferroni correction being overly conservative. The remaining methods perform well, S5M having a slight but significant (*P *<* *0.005 Welch’s *t*-test; see Welch (1947)) edge over its two-step competitor that is subsequently investigated on real-world data.

### 4.3 Real-world data experiment for onset of sepsis in the ICU on MIMIC-III

We compare S5M to the state-of-the-art comparison partner S3M for temporal pattern mining by extracting shapelets of vital signs of intensive care patients that are statistically significantly associated with the onset of Sepsis-3 (Singer *et al.*, 2016) in the MIMIC-III (*Multiparameter Intelligent Monitoring in Intensive Care*) database (Johnson *et al.*, 2016). Our experimental setup follows for the most part the same pipeline as in Bock *et al.* (2018), also using the queries from Johnson *et al.* (2018). The change to the pipeline is to not divide the dataset into training and testing parts, but to find associations on the full MIMIC-III cohort. In general significant pattern mining does not use a dataset split because the *P*-value is constructed with the assumption of a limited dataset (Pržulj, 2019, Chapter 8).

We search for statistically significant shapelets on heart rate, respiratory rate and systolic blood pressure associated with onset of sepsis comparing S5M to the internal comparison partner Tarone submodular and the state-of-the-art comparison partner S3M (Bock *et al.*, 2018). The number of shapelets returned by the three methods is shown in Table 1. Please recall that our goal is to use shapelets as a method for biomarker discovery for downstream applications. S3M returns a number of shapelets that is too large to process by humans and contains many redundant sequences. That is expected, because the approach is fundamentally underpowered due to not accounting for representativeness of the shapelets. Regarding the internal comparison, S5M returns a few more shapelets compared to its two-stage sibling approach Tarone submodular which is consistent with the assumption that S5M is of greater statistical power.

**Table 1.** The three comparison partners return a different number of statistically significant shapelets for temporal patterns in vital signs associated with Sepsis in MIMIC-III

Vital sign	S5M	Tarone submodular	S3M
Heart rate	45	42	11 895
Within (41 130)	25	40	10 548
Below/above 41/130	15/6	0/1	400/870
Respiratory rate	24	21	72 158
Within (9, 20)	0	0	0
Below/above 9/20	14/24	1/21	2047/72 050
Systolic blood pressure	56	41	104 863
Within (91 219)	0	22	58 342
Below/above 91/219	56/0	19/0	42 472/30

*Note*: Also shown is the number of shapelets within, below and above bounds that are associated with low clinical risk according to the national early warning score (McGinley and Pearse, 2012).

In Table 1, we also show the number of shapelets that lie in bounds that are associated with low clinical risk according to the national early warning score (McGinley and Pearse, 2012), a widely used scoring system for recognizing critical illness (Bersten and Handy, 2013). We observe fewer shapelets of S5M lying within a *normal* range, in contrast to Tarone submodular and S3M. Assuming that instability of vital signs is strongly associated with the patient at the onset of sepsis [which has been the hypothesis of Bock *et al.* (2018)], we conclude S5M yields the lowest number of false positives. Moreover, we find many shapelets which fall below the normal range. At first glance, this seems counter-intuitive because the human body is known to compensate for a state of shock by increasing respiratory rate and heart rate. On second glance, shapelets that show the vital sign falling below the normal range could indicate a failure of this compensation mechanism (or clinically: a decompensation) in patients with sepsis. Many shapelets of S5M exhibit this pattern, whereas only some shapelets in Tarone submodular do, including the shapelets found on respiratory rate. To conclude, S5M returns a more diverse set of shapelets that are at the same time more informative for patients with sepsis.

**Fig. 4.**
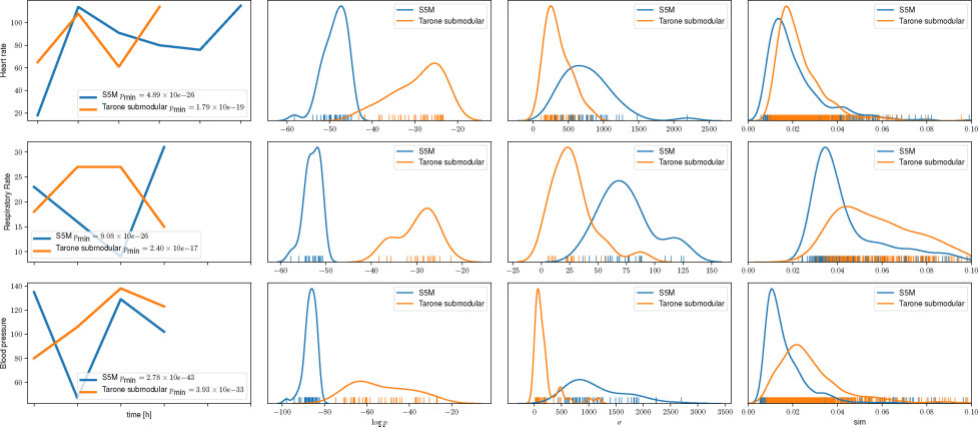
Comparing the extraction of representative and statistically significant shapelets associated with Sepsis in MIMIC-III with S5M and the two-step approach Tarone submodular. Each row refers to one clinical variable (heart rate, respiratory rate and systolic blood pressure). In the first column, the shapelet returned by S5M/Tarone submodular with the lowest *P*-value is depicted. Note that the particular date at which a shapelet occurs within a time series has no associated biological meaning, leaving the x-axis ticks blank. The second column shows the distribution of the shapelet *P*-values (on a log ⁡P-scale). In the third column, the structural diversity of the set of shapelets is shown by assessing the distribution of within-shapelet variances. The fourth column displays the distribution over the pairwise shapelet similarities. We observe that S5M returns a structurally more diverse set of shapelets with on average lower *P*-values including the shapelet with the lowest *P*-value, which was not deemed representative in Tarone submodular

We proceed to quantitatively compare the shapelets of S5M to the shapelets of its internal comparison partner Tarone submodular (Fig. 4). Each row corresponds to one clinical variable (heart rate, respiratory rate and systolic blood pressure). In the first column, the shapelet with the lowest *P*-value from both methods is shown. S5M consistently yields the shapelet with the lowest *P*-value, which was deemed non-representative in Tarone submodular. The second column shows the Gaussian kernel density estimate (Seabold and Perktold, 2010) of the distribution of the shapelet *P*-values (on a log ⁡p-scale). The distribution of *P*-values retrieved by S5M is more skewed towards lower *P*-values compared to Tarone submodular, which is consistent with the hypothesis that S5M has more statistical power. In the third column, the distribution of structural diversity is shown by looking at the within-shapelet variances. We can observe that S5M consistently yields shapelets that are of higher structural diversity. In the fourth column, the distribution of the pairwise similarities are shown. For heart rate, the results for Tarone submodular and S5M agree. For the other two vital signs, S5M yields on average lower pairwise similarities compared to Tarone submodular.

To summarize, the meaningful search for representatives should always be a stage in data mining driven temporal biomarker discovery. The only state-of-the-art competitor that allows for statistical significance testing, S3M, is fundamentally underpowered due to the very high number of shapelets. Moreover, we find the simultaneous approach (S5M) has more power yielding a structurally more diverse set of shapelets and fewer false positives compared to a post-processing approach of S3M.

### 4.4 Real-world data experiment for in-ICU mortality from the SOFA score on eICU

**Fig. 5.**
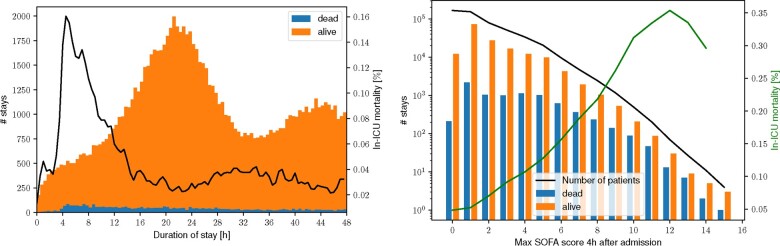
Left: proportion of patient stays exiting the ICU dead (blue) or alive (orange) as a distribution over the length of the patient stay. In black, the fraction of lethal stays is shown. The relative and absolute number of patients that exit the ICU dead sharply increases after 4 h. Right: admissions are categorized according to their maximum SOFA score during the first 4 h of the stay (*x*-axis). The number of stays is separated by patient outcome (dead or alive, *y*-axis). The green line shows the percentage of in-ICU mortality given the maximum SOFA score during the first four hours, which increases with increasing maximum SOFA score, reaching a plateau at a score of 10

The eICU collaborative research database for critical care (Pollard *et al.*, 2018) is the largest openly accessible critical care database available to date by patient numbers. Extracting the in-ICU mortality of patients in eICU, we notice an increase in patients dying in the ICU after their first four hours of stay (see Fig. 5 left).

The SOFA describes the severity of a patients organ dysfunction, developed for defining Sepsis (Singer *et al.*, 2016) but also used as a possible predictor of death in the ICU (Vincent *et al.*, 1996). The medical intuition of the SOFA score, in terms of organ dysfunction, is that it reflects the severity of critical illnesses. We have extracted the SOFA score for all 200 000 eICU patient admissions at an hourly resolution by forward filling each measurement and assigning the time of measurement to the next full hour (we also use urine output added to the database as recently as 2019). Note that previous studies assessing serial evaluations of SOFA used 48 h intervals or more (Ferreira *et al.*, 2001; Tee *et al.*, 2018). The high resolution of eICU measurements, however, makes more frequent assessments of the SOFA scores possible. We observe that with an increasing maximum SOFA score, the ICU mortality rate also increases, reaching a plateau at a SOFA score of 10 (see Fig. 5 right).

**Fig. 6.**
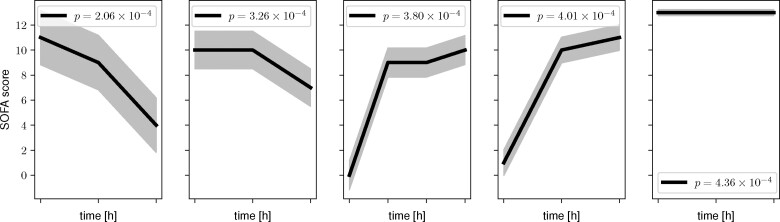
We show that the shapelets associated with in-ICU mortality conditioned on a maximum SOFA score larger than 10 within 4 h of admission are gridded to an hourly resolution. Note that the first two shapelets are associated with no in-ICU mortality, whereas the other three are associated with in-ICU mortality. The particular date at which a shapelet occurs within a time series has no associated biological meaning, leaving the *x*-axis ticks blank

We employ S5M on the first 4 h after admission for the 2600 stays with a maximum SOFA score of 10 or higher within the first 4 h (1954 controls, 546 death cases, length of time series *m *=* *5). The resulting statistically significant and representative shapelets returned by S5M for (not) dying in the ICU, conditioned on having a maximum SOFA score of 10 or higher in the first 4 h, are shown in Figure 6. The shaded area illustrates the threshold associated with that shapelet by indicating the region of SOFA score trajectories that are associated with the patient outcome. Note that since the SOFA score can only take discrete values, the pruning mechanisms of S3M fail when repeatedly testing the same (significant) subsequence which leads to an increase in runtime, making the comparison to S3M impossible.

The shapelets retrieved by S5M indicate that a SOFA score improvement (decrease) by six points within three hours is statistically significantly associated with the patients surviving the ICU stay. A sharp increase from 0 followed by a plateau at a SOFA score of 10 is statistically significantly associated with the patient dying in the ICU. Moreover, a constant SOFA score at 13 is also statistically significantly associated with a patient dying in the ICU. All three observations are in line with the clinical intuition that the SOFA score reflects the severity of critical illnesses. This serves as a proof of concept that S5M is a good method for biomarker discovery in time series datasets.

Conventionally, the SOFA score is evaluated only once upon admission and possibly followed up with on a daily basis in the ICU. In this work we first discover that for a cohort of high SOFA score, temporal patterns of the SOFA score during the first four hours of stay (as retrieved via S5M) can be statistically significantly associated with in-ICU mortality. This is a medically interesting finding, as it might help ICU practitioners to stratify and prioritize critically ill patients *soon* after ICU admission. To which degree our approach will show clinical utility and external validity will be an exciting route for future work.

## 5 Conclusion

This work introduced S5M for association mapping of interpretable temporal patterns in biomedical time series. The proposed method searches for subsequences that are both representatives for the set of all subsequences *and* statistically significantly associated with patient phenotype. S5M addresses a fundamental shortcoming of state-of-the-art temporal pattern mining approaches by optimizing a submodular mixture objective function that maximizes coverage and minimizes redundancy of patterns. Applying S5M to a time series dataset results in a set of temporal patterns that can be controlled in cardinality while achieving maximal structural diversity. By contrast, in traditional settings (in particular in classification scenarios), interpretability is not guaranteed due to the lack of a significance test or due to an uncontrollably large number of redundant shapelets and low statistical power.

These conceptual advances are primarily driven by a novel iterative optimizer for submodular optimization problems that was shown to have a lower runtime while yielding qualitatively and quantitatively comparable results to its traditional state-of-the-art competitors.

We have shown in both simulated and previously analyzed real-world datasets that the superiority of S5M in power is due to its capability of simultaneously pruning non-testable and non-representative patterns. Moreover, we discovered biomarkers in the SOFA score of ICU patients that are associated with in-ICU mortality that have a clear medical interpretation. In particular, our findings suggest a change in the assessment frequency of the SOFA score for clinical practitioners soon after ICU admission. This demonstrates that S5M is a reliable method for temporal biomarker discovery in time series datasets.

## Funding

This project was supported by the grant #2017-110 of the Strategic Focal Area ‘Personalized Health and Related Technologies (PHRT)’ of the ETH Domain for the SPHN/PHRT Driver Project ‘Personalized Swiss Sepsis Study’ (T.G., C.B. and M.M.; grant awarded to K.B.) and the Alfried Krupp Prize for Young professors of the Alfried Krupp von Bohlen und Halbach-Stiftung (K.B.).

*Conflict of Interest*: none declared. 

## Data availability

The datasets analyzed in this study are available from physionet.org
